# Fever phobia: a comparison survey between caregivers in the inpatient ward and caregivers at the outpatient department in a children’s hospital in China

**DOI:** 10.1186/s12887-015-0475-8

**Published:** 2015-10-19

**Authors:** Lili Dong, Jiahui Jin, Yili Lu, Lili Jiang, Xiaoou Shan

**Affiliations:** Department of Pediatric, Second Affiliated Hospital of Wenzhou Medical University, 109 Xueyuan Road, Wenzhou, Zhejiang Province 325027 China; Children’s hospital of Zhengzhou, Zhengzhou, Henan Province 450018 China

**Keywords:** Fever, Fever phobia, Caregivers, Ward, Outpatient, Knowledge

## Abstract

**Background:**

Fever in children is one of the most common clinical symptoms and a chief complaint and a main reason that caregivers took the children to the outpatient service or admitted to hospital. Studies have found that the majority of parents surveyed at a hospital pediatric clinic held unrealistic and unwarranted concerns about fevers, first termed as ‘fever phobia’ by Schmitt in 1980. In the present study, we explore whether ‘fever phobia’ exists in Chinese caregivers and investigate whether such phobia is alleviated when admitted to hospital after propaganda of fever related knowledge by doctors and nurses.

**Methods:**

A questionnaire was distributed to caregivers of children who visited the pediatric outpatient department and those with caregivers in the wards between June 2012 and Feb 2013 in Wenzhou, China.

**Results:**

Data were obtained from 621 caregivers, 305(49 %) from the OPD and 316(51 %) from the ward. Most caregivers of the two groups (OPD vs. ward group, 75.1 vs. 74.4 %) believed fever could cause brain damage. 77.7 % (76.0 vs. 81.3 %) caregivers were very worried when their children had fever and 12.8 % (14.1 vs. 11.4 %) caregivers would check the temperature within 30 min. Moreover, 68.0 % (63.0 vs. 72.8 %, *P* < 0.05) caregivers would give their children antipyretics during sleep and 39.9 % (40.3 vs. 39.6 %) would administrate antipyretics when temperature was above 38 °C. After admitted to hospital, 83.9 % caregivers stated to have received education about fever and 96.5 % felt relieved. Less caregivers (ward group vs. OPD, 42.4 vs. 46.9 %, *P* < 0.05) from ward group would give antipyretics with a temperature under 38.5 °C and less (0.6 vs. 4.9 %, *P* < 0.05) preferred cold sponging as physical cooling method compared to the OPD caregivers. Alarmingly, more caregivers (42.7 vs. 34.3 %, *P* < 0.05) in the ward group believed fever could lead to death or/and deafness (17.4 vs. 10.5 %, *P* < 0.05) and even 0.6 % caregivers in the ward group chose aspirin when the children had fever.

**Conclusion:**

‘Fever phobia’ also exists in Chinese caregivers. Fever related knowledge propaganda after admitted to hospital did not work effectively to improve the caregivers’ understanding and management of fever and an effective way to alleviate ‘Fever phobia’.

## Background

Fever is very common in children, usually indicating an underlying viral infection which is often harmless and self-limiting, while fever is sometimes a response to severe bacterial infection requiring timely and appropriate medical intervention [1], and always leading to a frequent concern for parents and caregivers [2, 3]. Parents and caregivers often misinterpret fever as being dreadful and harmful to their children, and believed that fever could cause brain damage or even death [4–6]. Fear of fever may sometimes results in frequent visits to the emergency department or clinic unnecessarily, thus leading to unscheduled physician visits, inappropriate treatments and unexpected financial budget [7]. Such misconception about fever has been coined as “fever phobia” by Schmitt in 1980 [4]. Since Schmitt’s study of “fever phobia” in caregivers, similar studies have been conducted in many other countries to describe the unrealistic fears about fever in both caregivers and healthcare professionals [8, 9]. Though more than 20 years has passed after the initial study, fever phobia still exists in the United States as well as in the other countries [10–16].

The wide existence of ‘Fever phobia’ reveals that the majority of caregivers think fever is harmful to their children and regard it as a disease rather than a symptom or sign of illness [4, 17–19], which explains the fear of fever and further intensifies the popularity of ‘Fever phobia’. In China, we found that some caregivers even administer antipyretics without doctors’ advice and ask pediatricians for antibiotic infusion for the purpose of rapid reduction of temperature, reflecting that ‘Fever phobia’ also exists in China and it may be more severe among caregivers. Besides, physical cooling methods are also included, such as undressed, tepid sponging, cold sponging, cold toweling, alcohol sponging or wine sponging. Actually, fever is a natural adaptive process which may improve the body’s resistance to future infections and the frequent use of antipyretics to artificially reduce the body temperature may inhibit this process [20]. Moreover, in children with febrile illness, no evidence has been found that the height of temperature correlates with the severity of illness except for that in very young children [21] and no evidence suggests that fever reducing improves morbidity or mortality [22]. Studies have shown that physical methods of temperature reduction do not treat the cause of fever and may cause discomfort, shivering, resulting in vasoconstriction and an increase in temperature and metabolism [23–25], caregivers still ignorantly apply them.

According to previous studies, education in the waiting place could help caregivers know well about fever and deal with it appropriately [26–29]. To the best of our knowledge, although many reports related to ‘Fever phobia’ have been conducted, no studies about the difference between the outpatient department (OPD) group and the ward group have been conducted. Among most hospitals in China, education by doctors or nurses about related diseases takes place after admission in the hospital.

Our objectives for this study is to explore caregivers’ attitudes towards fever and compare the attitudes between the OPD group and the ward group, and to determine whether the caregivers feel relieved and whether the education works after admitted to hospital.

## Methods

### Design

A cross-sectional survey was conducted as face-to-face interview between June 2012 and Feb 2013 in Wenzhou, China, which is a city that lies in the middle of east coast of China, southeast of Zhejiang province, and it is the economic, cultural and transportation center of Zhejiang. We selected a convenience sample of patients who were available and consented to be in the study while waiting at the OPD, and those with caregivers in the wards. All the respondents in the ward group had been educated for fever-related knowledge when interviewed. Children less than 3 months old and caregivers with experience of working in healthcare institutions and those who had the experience of hospitalization were excluded. According to Chinese Tradition, the questionnaire was re-administrated after Schmitt’, and was validated using content validity by an expert panel that comprised a senior clinical pediatrician, a senior clinical nurse and clinical educators. It consists of demographic information (e.g. patients’ age, place of residence) and 18 questions including caregivers’ knowledge about fever, causes of fever, possible effects of fever, concerns about fever, fever management, and source of information about fever. Additional information was collected from caregivers in the ward as to whether caregivers felt relieved, and received education from doctors and nurses after admitted to hospital. Caregivers gave verbal and written consent. The Second Hospital of Wenzhou Medical University Research Ethics Committee approved the study.

### Statistical analysis

Data analysis was performed with SPSS 19.0 for Windows. Student’s *t* test was used for continuous variables; *χ*^2^ analysis was used for dichotomous variables. If the *p* value was <0.05, it was considered significant.

## Results

### Demographics

Of all the 621 caregivers, 316 (51 %) from the ward and 305 (49 %) from the OPD agreed to be in the survey and were interviewed. The ward group and the OPD group differed demographically by both ‘caregiver’s occupation’ and ‘place of residence’ (Table 1). Regarding to caregiver’s occupation, in the ward group, 33.9 % were house wives or had no job, 32.0 % self-employed, 14.2 % workmen, 10.4 % institutional organization workers and 8.2 % worked for enterprise, while in the OPD, the most were workmen, accounting for 34.2 %, followed by 27.8 % caregivers who had no job, 19.9 % self-employed, 10.3 % working for enterprise and 6.4 % institutional organization workers, separately (*p* < 0.05). Caregivers living in countryside accounted for 59.2 % in both of the two groups, while 21.5 % from the ward group lived in the city and those who lived in the city from the OPD group accounted for 27.8 % (*p* < 0.05). The remaining demographic variables were comparable between groups. Mothers accounted for 76.0 % of those interviewed, and 66.7 % of those interviewed were between the ages 26 and 35. Among those interviewed, 36.7 % graduated from middle school, 25.6 % from college, 22.4 % from high school, 10.6 % from primary school and 1.4 % received no education. Most of those interviewed (80.7 %) were local residents, and 55.1 % of the caregivers interviewed had only one child, 38.8 % had 2, while 6.1 % had three or more. At the time of interview, the family annual income was $5000 or less (23.2 %), $5000–8000 (32.9 %), $8000–16,000 (26.9 %) or $16,000 or more (17.1 %). (All the data above were shown in Table 1).

**Table 1:** Distribution of demographic characteristics

Variables	Total	OPD	Ward	*p* value
	*n* = 621 (%)	*n* = 305 (%)	*n* = 316 (%)	
Caregivers				
Mothers	472 (76.0)	221 (72.5)	251 (79.4)	
Fathers	126 (20.3)	73 (23.9)	53 (16.8)	
Grandparents	21 (3.4)	11 (3.6)	12 (3.8)	0.085
Caregiver’s age, years				
25 or younger	88 (14.2)	49 (16.1)	39 (12.3)	
26–35	414 (66.7)	193 (63.3)	221 (69.9)	
36–45	103 (16.6)	57 (18.7)	46 (14.6)	
46 or older	16 (2.6)	6 (2.0)	10 (3.2)	0.171
Caregiver’s occupation				
Unemployed (House wife)	191 (30.8)	84 (27.8)	107 (33.9)	
Workman	149 (24.0)	104 (34.2)	45 (14.2)	
Self-employed	162 (26.1)	61 (19.9)	101 (32.0)	
Enterprise	58 (9.3)	32 (10.3)	26 (8.2)	
Institutional organization workers	53 (20.3)	20 (6.4)	33 (10.4)	
Government officer	8 (1.3)	4 (1.4)	4 (1.3)	<0.001
Educational level				
Illiterate	9 (1.4)	3 (1.1)	6 (1.9)	
Primary school	66 (10.6)	28 (9.8)	38 (12.0)	
Middle school	228 (36.7)	120 (42.1)	108 (34.2)	
High school	139 (22.4)	56 (19.6)	83 (26.3)	
College	159 (25.6)	78 (27.4)	81 (25.6)	0.143
Native place				
Wenzhou, Zhejiang	501 (80.7)	249 (81.6)	252 (79.7)	
Non Wenzhou	120 (19.3)	56 (18.4)	64 (20.3)	0.550
Place of residence				
Countryside	367 (59.1)	180 (59.2)	187 (59.2)	
County town	101 (16.3)	40 (13.0)	61 (19.3)	
City	153 (24.6)	85 (27.8)	68 (21.5)	0.045
Number of children				
1	342 (55.1)	178 (58.4)	164 (51.9)	
2	241 (38.8)	112 (36.7)	129 (40.8)	
3 or more	38 (6.1)	15 (4.9)	23 (7.3)	0.196
Annual income of the family				
$5000 or less	144 (23.2)	65 (21.4)	79 (24.9)	
$5000–8000	204 (32.9)	107 (35.0)	97 (30.7)	
$8000–16,000	167 (26.9)	80 (26.3)	87 (27.6)	
$16,000 or more	106 (17.1)	53 (17.3)	53 (16.9)	0.583

### Definition of fever and high fever

When asked about the definition of fever, 9.8 % caregivers (10.2 vs. 9.5 %) considered 37 °C as the threshold of fever, 39.8 % considered 37.5 °C, and a large number of caregivers considered 38 °C. Except one in the ward group who did not know, the rest defined the temperature below 38 °C as fever. Moreover, the most common high fever threshold was 39 °C, respectively reported by 55.4 % and 57.3 % caregivers, then, followed by 38.5 °C (26.6 vs. 27.8 %) and 40 °C (6.2 vs. 8.9 %). While the two groups did not differ statistically on the definition of fever and high fever, and data was specifically illustrated in Table 2.

**Table 2:** Definition of fever and high fever by caregivers

Variables	Total	OPD	Ward	*p* value
	*n* = 621 (%)	*n* = 305 (%)	*n* = 316 (%)	
Body temperature considered as fever (°C)				
≥ 37	61 (9.8)	31 (10.2)	30 (9.5)	
≥ 37.5	247 (39.8)	118 (38.7)	129 (40.8)	
≥ 38	312 (50.2)	156 (51.1)	156 (49.4)	
Unknown	1 (0.2)	0 (0.0)	1 (0.3)	0.726
Body temperature considered as high fever (°C)				
≥ 37	1 (0.2)	1 (0.3)	0 (0.0)	
≥ 38	53 (8.5)	35 (11.5)	18 (5.7)	
≥ 38.5	169 (27.2)	81 (26.6)	88 (27.8)	
≥ 39	350 (56.4)	169 (55.4)	181 (57.3)	
≥ 40	47 (7.6)	19 (6.2)	28 (8.9)	
Unknown	1 (0.2)	0 (0.0)	1 (0.3)	0.085

### Knowledge about fever

With respect to the knowledge about fever, about 2.8%caregivers in the ward group would give antipyretics at the temperature of 37 °C, 39.6 % at the temperature of 38 °C, 47.2 % at the temperature of 38.5 °C, 10.4 % at the temperature of 39 °C; While in the OPD, the percentage of caregivers administrating antipyretics at each temperature above were 6.6, 40.3, 44.9, 7.2 %, respectively. Moreover, three caregivers in this group had even no idea. It was of statistical significance between the two groups.

The most common antipyretics chosen by caregivers were ibuprofen, and the proportion in the ward group and the outpatient group were 78.2 and 80.7 % separately. The percentage of caregivers (9.5 %) who would use acetaminophen were the same between the two groups. And the method of combining or alternating ibuprofen and acetaminophen was also seen in 4.7 % caregivers in the ward group and 3.9 % in the OPD group. While 2(0.6 %) caregivers in the ward group chose aspirin. The left 7.0 % in the ward group and 5.9 % in the outpatient group had other choices, mostly were Chinese traditional medicine, such as Radix isatidis (banlangen), Qingkailing granules (qingkailingkeli), Taurine Granules and Radix Bupleuri Oral Liquids (Chaihukoufuye), and others included Compound Zinc Gluconate and Ibuprofen Granules and suppositories. And most of the caregivers, 63.0 % in the outpatient group would awaken their febrile sleeping children for antipyretics administration, while those in the ward group were significantly more (*p* < 0.05) and the percentage was up to 72.8 %.

With regard to the way of getting antipyretics, 38.3 % caregivers in the ward group and 29.2 % in the outpatient group went to drugstores, but hospital was still the major choice, 56.3 % in the ward group and 66.6 % in the outpatient group, while 5.4 % in the ward group and 4.3 % in the outpatient group would go to both drugstore and hospital, as shown in Table 3 and there was statistical significance between the two groups (*p* < 0.05).

**Table 3:** Knowledge about fever

Variables	Total	OPD	Ward	*p* value
	*n* = 621 (%)	*n* = 305 (%)	*n* = 316 (%)	
Body temperature considered to give antipyretics (°C)				
≥ 37	29 (4.7)	20 (6.6)	9 (2.8)	
≥ 38	248 (39.9)	123 (40.3)	125 (39.6)	
≥ 38.5	286 (46.1)	137 (44.9)	149 (47.2)	
≥ 39	55 (8.9)	22 (7.2)	33 (10.4)	
Unknown	3 (0.5)	3 (1.0)	0 (0.0)	0.046
Antipyretics used				
Ibuprofen	493 (79.4)	246 (80.7)	247 (78.2)	
Acetaminophen	59 (9.5)	29 (9.5)	30 (9.5)	
Ibuprofen &Acetaminophen	27 (4.3)	12 (3.9)	15 (4.7)	
Aspirin	2 (0.3)	0 (0.0)	2 (0.6)	
Banlangen^a^	1 (0.2)	0 (0.0)	1 (0.3)	
Qingkailingkeli^b^	3 (0.5)	1 (0.2)	2 (0.6)	
Chaihukoufuye^c^	2 (0.3)	1 (0.2)	1 (0.3)	
Compound Xinbu Granules^d^	3 (0.5)	1 (0.2)	2 (0.6)	
Niuhuangsuankeli^e^	3 (0.5)	1 (0.2)	2 (0.6)	
Xiaoerqingrejiekeli^f^	3 (0.5)	1 (0.2)	2 (0.6)	
Unknown	25 (4.0)	13 (4.3)	12 (3.8)	0.634
Giving antipyretics during sleep				
Yes	422 (68.0)	192 (63.0)	230 (72.8)	
No	199 (32.0)	113 (37.0)	86 (27.2)	0.009
Where to get antipyretics				
Drugstore	210 (33.8)	89 (29.2)	121 (38.3)	
Hospital	381 (61.4)	203 (66.6)	178 (56.3)	
Drugstore & Hospital	30 (4.8)	13 (4.3)	17 (5.4)	0.032
The method of physical cooling				
Tepid sponging	287 (46.2)	136 (44.6)	151 (47.8)	0.469
Cold sponging	17 (2.7)	15 (4.9)	2 (0.6)	0.001
Cold toweling	93 (15.0)	75 (24.6)	108 (34.2)	0.01
Alcohol sponging	121 (19.5)	58 (19.0)	63 (19.9)	0.803
Fever cooling patch	65 (10.5)	27 (8.9)	38 (12.0)	0.197
Unknown/No	47 (7.6)	26 (8.5)	21 (6.6)	0.376
Wine	2 (0.3)	0 (0.0)	2 (0.6)	0.499

^a^Radix isatidis; ^b^Qingkailing granules; ^c^Taurine Granules and Radix Bupleuri Oral Liquids; ^d^Compound Zinc Gluconate and Ibuprofen Granules; ^e^Taurine Granules; ^f^Qingrejie granules

Even if there was not significant evidence showing any benefits of physical cooling, 47.8 % in the ward group and 44.6 % in the outpatient group, nearly half of the caregivers still used tepid sponging for their feverish children and there were significantly more caregivers choosing cold sponging (0.6 vs. 4.9 %, *p* < 0.05). While 2 caregivers even chose wine sponging. There were 34.2 % caregivers in the ward group and 24.6 % in the outpatient group choosing cold toweling, which was of statically significance (*p* < 0.05). In terms of those choosing alcohol sponging, the number was nearly the same, besides, there was the convenient method fever cooling patch preferred by 12.0 % in the ward group and 8.9 % in the outpatient group. Then there were 6.6 % caregivers in the ward group and 8.5 % in the outpatient group who had never used the methods of physical cooling or had no idea (shown in Table 3).

### Potential effects of fever

The most potential effects of fever predicted by caregivers was brain damage, which was up to 74.7 %, although there was no statistical significance between the two groups (75.1 vs. 74.4 %, *p* > 0.05). There were significantly more caregivers in the ward predicting that death was the second most possible complication of fever (34.4 vs. 42.7 %, *p* < 0.05). According to the data obtained, 21.9 % caregivers believed fever can cause convulsion though without difference between the two groups (22.6 vs. 21.2 %, *p* > 0.05). As for the loss of hearing, the percentage was obviously higher in the ward group and it was statistically significant (10.5 vs. 17.4 %, *p* < 0.05). In addition, 11.9 % caregivers (10.2 vs. 13.6 %) regarded blindness as another possible danger of fever, while a small number of caregivers (1.7 vs. 1.9 %) stated they “Have no idea” at all. Data were displayed in detail in Table 4.

**Table 4:** Potential effects of fever

Variables	Total	OPD	Ward	*p* value
	*n* = 621 (%)	*n* = 305 (%)	*n* = 316 (%)	
Brain damage	464 (74.7)	229 (75.1)	235 (74.4)	0.838
Death	240 (38.6)	105 (34.4)	135 (42.7)	0.034
Convulsion	136 (21.9)	69 (22.6)	67 (21.2)	0.669
Deafness	87 (14.0)	32 (10.5)	55 (17.4)	0.020
Blindness	74 (11.9)	31 (10.2)	43 (13.6)	0.185
Have no idea	11 (1.8)	5 (1.7)	6 (1.9)	0.806

### How often temperature checked and level of caregiver’s worrisome

A vast majority of caregivers took their febrile child’s temperature at least every 2 h or more frequently, 32.7 % checked temperature every 1 to 2 h, 33.3 % checked every 30 min to 1 h, 11.8 % did it every 15 to 30 min. While 1.0 % checked the temperature every less than 15 min, only 20.6 % checked more than 2 h. All the data obtained about frequency of checking temperature between two groups was of no statistical significance (*p* > 0.05). Among 621 caregivers surveyed, 7 caregivers stated they were not worried at all, while 44.4 % were very worried and 34.3 % were extremely worried. Again, they did not differ statistically (*p* > 0.05), as shown in Table 5.

**Table 5:** How often temperature checked and level of caregiver’s worrisome

Variables	Total	OPD	Ward	*p* value
	*n* = 621 (%)	*n* = 305 (%)	*n* = 316 (%)	
How often temperature checked				
≤ 15 min	6 (1.0)	5 (1.6)	1 (0.3)	
15–30 min	73 (11.8)	38 (12.5)	35 (11.1)	
30 min–1 h	207 (33.3)	98 (32.1)	109 (34.5)	
1 h–2 h	203 (32.7)	99 (32.5)	104 (32.9)	
≥ 2 h	128 (20.6)	63 (20.7)	65 (20.6)	
Unknown	4 (0.6)	2 (0.7)	2 (0.6)	0.648
Level of caregiver’s worrisome				
Not worried at all	7 (1.1)	2 (0.7)	5 (1.6)	
A little worried	125 (20.1)	71 (23.3)	54 (17.1)	
Very worried	276 (44.4)	137 (44.9)	139 (44.0)	
Extremely worried	213 (34.3)	95 (31.1)	118 (37.3)	0.116

### Sources for information about fever

Doctors and nurses were considered to be the primary source for information about fever, and the percentage was significantly higher in the ward group than that in the OPD group (53.3 vs. 43.0 %, *p* < 0.05). Compared with the OPD group, more caregivers chose Internet as their source for information about fever in the ward group. As to the experience, the proportion is statically higher in the ward group than that in the OPD group. While there was no difference between the two groups about the other sources for information of fever, such as TV or newspapers, professional lectures and neighbors or relatives, seen in Table 6.

**Table 6:** Sources for information about fever

Variables	Total	OPD	Ward	*p* value
	*n* = 621 (%)	*n* = 305 (%)	*n* = 316 (%)	
TV or newspaper	153 (24.6)	84 (27.5)	69 (21.8)	0.099
Parent’s books	179 (28.8)	87 (28.5)	92 (29.1)	0.871
Internet	139 (22.4)	58 (19.0)	81 (25.6)	0.048
Professional lectures	28 (4.5)	13 (4.1)	15 (4.7)	0.771
Neighbors or relatives	126 (20.3)	68 (22.3)	58 (18.4)	0.222
Doctors and nurses	300 (48.3)	131 (43.0)	169 (53.5)	0.009
Experience	155 (25.0)	45 (14.8)	110 (34.8)	<0.001

## Discussion

Fever is defined as ‘an elevation of body temperature above the normal daily variation, usually take the body temperature 38 °C or higher as a criteria. It’s the most common reason that caregivers took their children to doctors, accounting for almost one third of all the causes for children seeking medical advice [30–32]. However, the majority caregivers thought fever harmful to their children and considered it as a disease rather than a symptom or a sign of illness and necessary to be treated, thus leading to parents waking up their children to give antipyretics, in the pursuit of reducing to normal temperature and unimaginable and unrealistic worrisome about fever, namely ‘Fever phobia’ [4, 9, 33–36].

In our study, 9.8 % caregivers considered 37 °C as the threshold of fever, 39.8 % considered 37.5 °C, and the rest 50.2 % of caregivers considered 38 °C in the OPD. Except one in the ward group who did not know, the rest defined the temperature below 38 °C as fever. And the most common high fever threshold was 39 °C, reported by 55.4 and 57.3 % caregivers, respectively, then followed by 38.5 °C. But the less common high fever threshold was 40 °C. Other studies have reported similar findings. In Chang’s study, 81 % of participants considered temperature below 38 °C as the threshold of fever and 69.6 % considered a temperature below 39 °C as the threshold of high fever [37]. In Karwowska’s study, parents considered a temperature 37.9 °C as the threshold of fever and 39.1 °C as high fever [13]. In Poirier’s study, 55 % parents considered a temperature below 37.8 °C as the threshold of fever and 17.5 % as a temperature below 38.9 °C as high fever [38]. After admitted to hospital, though 83.9 % caregivers stated to have received education about fever and 96.5 % said to feel relieved, between the two groups, there was no significant difference as to the definition of fever and high fever at all, moreover, no difference was found about the antipyretics used between the two groups as well. It seemed that caregivers in China mainland had the same level of misunderstanding about the definition of fever as caregivers from other countries and the education after admission to hospital did not work effectively indicating that the education might did not refer to the related knowledge, or the education was not accepted, or even the related knowledge was rightly educated.

Among previously reported studies, 56 % parents were very worried about the possible harm caused by childhood fever in United States [34], 50–60 % in a Singaporean research [39], 65 % in a UK one [15], and 86.8 % parents in Taiwan stated to be worried [37]. In the present study, we found that 44.4 % caregivers were very worried and 34.3 % were even more worried. And 57.2 % would take their febrile children to hospitals at night, 74.7 % thought fever could cause brain damage, 4.7 % would give antipyretics when the temperature was higher than 37 °C and 38.0 % would choose 38 °C as threshold for antipyretics, 68.0 % would wake up their sleeping children for antipyretics, indicating a deep misunderstanding and unrealistic worrisome of fever, which was consistent with previous studies [9, 34, 40]. Such misunderstanding and worrisome of fever resulted in inappropriate or unnecessary treatments, thus leading to unexpected harm to children. Besides, there were no significant differences between the two groups in the above items, except that more caregivers in the ward group would wake up their sleeping children for antipyretics and thought deafness was one of the most potential effects of fever, suggesting the reason why the caregivers receiving inpatient care could be more concerned is the possibility of their child’s more severe disease. Moreover, those caregivers in the ward group might think that the fever would persist or get worse and could lead to more serious damage to children, which also reflected that the education after admission to the hospital did not work.

In previous studies, many kinds of harmfulness thought by caregivers were related to fever, the most common were convulsion, brain damage [4, 34, 39] and even death [4–6]. Crocetti et al. found that 53 % caregivers thought fever would rise if left untreated and 58 % thought fever itself could lead to brain damage or even death [34]. While in our study, in the outpatient group, 75.1 % thought fever could lead to brain damage, 34.4 % thought it could lead to death, 22.6 % thought it could lead to convulsion, 10.5 % thought it could lead to deafness and 10.2 % thought it was blindness, while in the ward group, the percentage was 74.4, 42.7, 21.2, 17.4, 13.6 % respectively, consistent with the previous study, Enarson et al. found 74 % parents thought fever dangerous and 90.3 % tried to reduce temperature [35]. The first three potential effects of fever predicated by caregivers were brain damage, death and convulsion, which may be the major reason why caregivers felt so worried about the fever and were in the pursuit of reducing to normal temperature. In fact, fever in children were mostly caused by virus [21, 41, 42], and if the children were with normal spirits and had sufficient water without dehydration, it would disappear without treatment.

Moreover, there are a number of physical cooling methods used to reduce body temperature, including undressing, sponging or toweling with tepid or cold water or alcohol, and these are involved in convection and evaporation but do not treat the underlying causes of the fever and the physical methods can cause discomfort [43, 44], such as shivering, hypoglycemia, coma and even death [4]. In our survey, though more caregivers in the ward group chose cold toweling and less chose cold sponging, there were equivalent caregivers in two groups choosing tepid sponging, alcohol sponging, fever cooling patch, and far more less caregiving did not know what physical cooling methods or did not put that into use, indicating that the education by doctors and nurses after admitted to hospital did not work as no physical interventions for reducing temperature were recommended in the recent published guideline for feverish illness for children [20].

Our findings underline the existing of ‘Fever phobia’ in Wenzhou, China but not the value of education after admission to a hospital in order to modify the caregivers’ understanding and management of fever. Caregivers took doctors and nurses as the first choice of source of information about fever, while there was no statistical significance between the two groups in most items, indicating that the education might not work effectively. Though previous education [45–47] showed positive effects, here it did not show fever-related education an effective action to improve the caregivers’ understanding and management of fever and an effective way to alleviate ‘Fever phobia’. Thus there are yet a lot that we can do to it.

### Limitations

The findings of this study should be considered within the following limitations. The study was undertaken at one pediatric hospital and the sample was small thereby limiting the generalisability of other findings and probably not being able to totally represent the national data. The caregivers interviewed had no experience of hospitalization, limiting the findings of the understanding of fever in those with the experience of hospitalization. We also care about the external validity of our findings relating to the perceptions of doctors and nurses. Although the doctors and nurses involved in the ward group survey have a variety of training backgrounds, knowledge base and perspective may differ when educating caregivers. Besides, there is also the possibility that caregivers in ward group might be more concerned due to their child’s more severe disease. Here, the reasons for outpatient visits and hospitalization were not studied and analyzed, which we will do during the next related study.

## Conclusions

“Fever phobia” is still widespread among caregivers and the vast majority believes that fever is harmful and it also exists in Chinese caregivers. In our study, it did not show fever education an effective action to improve the caregivers’ understanding and management of fever and an effective way to alleviate ‘Fever phobia’. Considering that there was no statistical significance between the two groups in some items, and both caregivers in the ward group and in the OPD group, doctors and nurses were considered as the primary source of information, underlying possible problems with the education, which suggested the importance to guarantee that doctors and nurses give parents correct and consistent information in order to eliminate “fever phobia”, some related training might be necessary.
